# Sentiment Analysis in Health and Well-Being: Systematic Review

**DOI:** 10.2196/16023

**Published:** 2020-01-28

**Authors:** Anastazia Zunic, Padraig Corcoran, Irena Spasic

**Affiliations:** 1 School of Computer Science & Informatics Cardiff University Cardiff United Kingdom

**Keywords:** sentiment analysis, natural language processing, text mining, machine learning

## Abstract

**Background:**

Sentiment analysis (SA) is a subfield of natural language processing whose aim is to automatically classify the sentiment expressed in a free text. It has found practical applications across a wide range of societal contexts including marketing, economy, and politics. This review focuses specifically on applications related to health, which is defined as “a state of complete physical, mental, and social well-being and not merely the absence of disease or infirmity.”

**Objective:**

This study aimed to establish the state of the art in SA related to health and well-being by conducting a systematic review of the recent literature. To capture the perspective of those individuals whose health and well-being are affected, we focused specifically on spontaneously generated content and not necessarily that of health care professionals.

**Methods:**

Our methodology is based on the guidelines for performing systematic reviews. In January 2019, we used PubMed, a multifaceted interface, to perform a literature search against MEDLINE. We identified a total of 86 relevant studies and extracted data about the datasets analyzed, discourse topics, data creators, downstream applications, algorithms used, and their evaluation.

**Results:**

The majority of data were collected from social networking and Web-based retailing platforms. The primary purpose of online conversations is to exchange information and provide social support online. These communities tend to form around health conditions with high severity and chronicity rates. Different treatments and services discussed include medications, vaccination, surgery, orthodontic services, individual physicians, and health care services in general. We identified 5 roles with respect to health and well-being among the authors of the types of spontaneously generated narratives considered in this review: a sufferer, an addict, a patient, a carer, and a suicide victim. Out of 86 studies considered, only 4 reported the demographic characteristics. A wide range of methods were used to perform SA. Most common choices included support vector machines, naïve Bayesian learning, decision trees, logistic regression, and adaptive boosting. In contrast with general trends in SA research, only 1 study used deep learning. The performance lags behind the state of the art achieved in other domains when measured by F-score, which was found to be below 60% on average. In the context of SA, the domain of health and well-being was found to be resource poor: few domain-specific corpora and lexica are shared publicly for research purposes.

**Conclusions:**

SA results in the area of health and well-being lag behind those in other domains. It is yet unclear if this is because of the intrinsic differences between the domains and their respective sublanguages, the size of training datasets, the lack of domain-specific sentiment lexica, or the choice of algorithms.

## Introduction

Sentiment analysis (SA), also known as opinion mining, is a subfield of natural language processing (NLP) whose aim is to automatically classify the sentiment expressed in a free text. Its origins can be traced to the 1990s including methods for classifying the point of view [1], predicting the semantic orientation of adjectives [2], subjectivity classification [3], etc. However, its rapid growth is correlated with the advent of Web 2.0 and the increasing availability of user-generated data such as product and service reviews as well as the proliferation of social media communication channels.

SA has found practical applications across a wide range of societal contexts including marketing, economy, and politics [4-8]. This review focuses specifically on applications related to health, which is defined as “a state of complete physical, mental, and social well-being and not merely the absence of disease or infirmity” [9]. The well-being itself is considered to be a perceived or subjective state, that is, it can vary considerably across individuals with similar circumstances [10]. This makes well-being an ideal case study for SA. However, when it comes to matters of health, modern society tends to be preoccupied with the negative phenomena such as diseases, injuries, and disabilities [11], which makes SA in this domain challenging. For instance, for a patient with a chronic condition, having a good quality of life will not necessarily depend on the absence of associated symptoms, but rather on the extent to which they are managed and controlled. However, the negative connotation of health symptoms tends to skew the SA results toward the negative spectrum.

To establish the state of the art in SA related to health and well-being, we conducted a systematic review of the recent literature. To capture the perspective of those individuals whose health and well-being are affected, we focused specifically on spontaneously generated content and not necessarily that of health care professionals. This differentiates this review from others conducted on related topics. For example, Denecke and Deng [12] reviewed SA in medical settings*,* but focused on the word usage and sentiment distribution of clinical data, such as nurse letters, radiology reports, and discharge summaries, while public data shared by the likes of patients and caregivers were restricted to 2 websites. On the contrary, Gohil et al [13] dealt with user-generated data, but only considered Twitter, whereas we posed no restrictions on the platforms used to generate the data.

The remainder of the paper is organized as follows. The Methods explains the methodology of this systematic review in detail. Results presents the findings of the review, followed by a discussion. The final section summarizes the main findings of the review.

## Methods

### Guidelines

Our methodology is based on the guidelines for performing systematic reviews described by Kitchenham [14]. It is structured around the following steps:

1. Research questions define the scope, depth, and the overall aim of the review.
2. Search strategy is an organized process designed to identify all studies that are relevant to the research questions in an efficient and reproducible manner.
3. Inclusion and exclusion criteria define the scope of a systematic review.
4. Quality assessment refers to a critical appraisal of included studies to ensure that the findings of the review are valid.
5. Data extraction is the process of identifying the relevant information from the included studies.
6. Data synthesis involves critical appraisal and synthesis of evidence to support the findings of the review.

### Research Questions

The overarching topic of this review is the SA of spontaneously generated narratives in relation to health and well-being. The main aim of this review was to answer the research questions given in Table 1.

**Table 1:** Research questions.

ID	Question
RQ1	What are the major sources of data?
RQ2	What is the originally intended purpose of spontaneously generated narratives?
RQ3	What are the roles of their authors within health and care?
RQ4	What are their demographic characteristics?
RQ5	What areas of health and well-being are discussed?
RQ6	What are the practical applications of SA^a^?
RQ7	What methods have been used to perform SA?
RQ8	What is the state-of-the-art performance of SA?
RQ9	What resources are available to support SA related to health and well-being?

^a^SA: sentiment analysis.

### Search Strategy

To systematically identify articles relevant to SA related to health and well-being, we first considered relevant data sources: the Cochrane Library [15], MEDLINE [16], EMBASE [17], and CINAHL [18]. MEDLINE was chosen as the most diverse data source with respect to the topics covered and publication types. MEDLINE is a premier bibliographic database that contains more than 29 million references to articles in life sciences and biomedicine. Its coverage dates back to 1946, and its content is updated daily. It covers publications of various types, for example, journal articles, case reports, conference papers, letters, comments, guidelines, and clinical trials. Its content is systematically indexed by Medical Subject Headings (MeSH), a hierarchically organized terminology for cataloging biomedical information, to facilitate identification of relevant articles. For example, it defines the term *natural language processing* as “computer processing of a language with rules that reflect and describe current usage rather than prescribed usage.” Therefore, this term can be used to identify articles on this topic even when they use alternative terminology, for example, “sentiment analysis,” “information retrieval,” and “text mining.” We used PubMed, a multifaceted interface, to search MEDLINE.

Having chosen MEDLINE as the primary source of information, the next step in developing our search strategy was to define a search query that adequately describes the chosen topic—SA related to health and well-being. Given the MEDLINE’s focus on biomedicine, inclusion of terms related to health and well-being was considered redundant. Specifically, they could improve the precision of the search (ie, reduce the number of irrelevant articles retrieved), but could only decrease the recall (the number of relevant articles retrieved). Given the relative recency of research into SA and its applications in biomedicine, we expected a query focusing solely on SA to retrieve a manageable number of articles, which could then be reviewed manually. The search query was defined as follows:

((sentiment[Title] OR sentiments[Title] OR opinion[Title] OR opinions[Title] OR emotion[Title] OR emotions[Title] OR emotive[Title] OR affect[Title] OR affects[Title] OR affective[Title]) AND (“sentiment classification” OR “opinion mining” OR “natural language processing” OR NLP OR “text analytics” OR “text mining” OR “F-measure” OR “emotion classification”)) OR “sentiment analysis”

The search performed on January 24, 2019, retrieved a total of 299 articles. Notably, no articles published before 2011 were retrieved, which confirmed our hypothesis about the relative recency of research into SA and its applications in biomedicine.

### Selection Criteria

To further refine the scope of this systematic review, we defined a set of inclusion and exclusion criteria (see Tables 2 and 3) to select the most appropriate articles from those matching the search query.

Two annotators independently screened the retrieved articles against inclusion and exclusion criteria and achieved the interannotator agreement of 0.51 calculated using Cohen kappa coefficient [19]. Disagreements were resolved by the third independent annotator. A total of 95 articles were retained for further processing.

**Figure 1:**
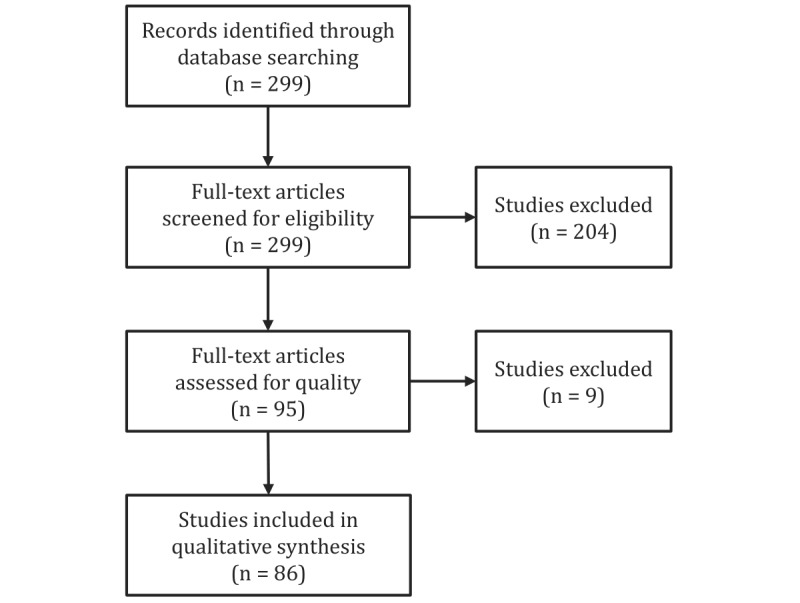
Flow diagram of the literature review process.

To ensure the rigorousness and credibility of selected studies, they were additionally evaluated against the quality assessment criteria defined in Table 4. A total of 9 studies were found not to match the given criteria. This further reduced the number of selected articles to 86. Figure 1 summarizes the outcomes of the 4 major stages in the systematic literature review.

**Table 2:** Inclusion criteria.

ID	Criterion
IN1	The input text represents spontaneously generated narrative.
IN2	The input text discusses topics related to health and well-being.
IN3	The input text captures the perspective of an individual personally affected by issues related to health and well-being (eg, patient or carer) rather than that of a health care professional.
IN4	Sentiment is analyzed automatically using natural language processing.

**Table 3:** Exclusion criteria.

ID	Criterion
EX1	Sentiment analysis is performed in a language other than English.
EX2	The article is written in a language other than English.
EX3	The article is not peer reviewed.
EX4	The article does not describe an original study.
EX5	The article is published before January 1, 2000.
EX6	The full text of the article is not freely available to academic community.

**Table 4:** Quality assessment criteria.

ID	Criterion
QA1	Are the aims of the research clearly defined?
QA2	Is the study methodologically sound?
QA3	Is the method explained in sufficient detail to reproduce the results?
QA4	Were the results evaluated systematically?

### Data Extraction and Synthesis

Data extraction cards were designed to aid the collection of information relevant to the research questions. They included items described in Table 5. The selected articles were read in full to populate the data extraction cards, which were then used to facilitate narrative synthesis of the main findings.

**Table 5:** Data extraction framework.

Item	Description
Data	Provenance, purpose, selection criteria, size, and use.
Topic	General topic discussed in the given dataset including medical conditions and treatments.
Author	Author (data creator) demographics and their role in health care.
Application	Downstream application of SA^a^ results.
Method	Type of SA method used, feature selection/extraction, and any resources used to support implementation of the method.
Evaluation	Measures used to evaluate the results, specific results reported, baseline method used, and improvements over the baseline (if any).

^a^SA: Sentiment analysis.

## Results

### Data Provenance

This section discusses the main properties of data used as input for SA in relation to research questions RQ1 and RQ2. The majority of data were collected from the mainstream social multimedia and Web-based retailing platforms, which provide the most pervasive user base together with application programming interfaces (APIs) that can support large-scale data collection. Not surprisingly, 26 studies [20-45] used data sourced from Twitter, a social networking service on which users post messages restricted to 280 characters (previously 140). Twitter can be accessed via its API from a range of popular programming languages using libraries such as TwitterR [22], Twitter4J in Java [29,41], and Tweepy in Python [45].

Facebook, another social networking service, was used to collect user posts regarding Chron's disease [46] and depression and anxiety [47]. Comments posted on Instagram, a photo and video-sharing social networking service, were used to predict depression [48]. A total of 2 studies used data from YouTube, a video-sharing website, which allows users to share videos and comment on them. These studies collected comments on videos related to proanorexia [49] and Invisalign experience [50]. Reddit, a social news aggregation, Web content rating, and discussion website, was used to learn to differentiate between suicidal and nonsuicidal comments [51]. Amazon, a Web-based retailer, allows users to submit reviews of products. Customers may comment or vote on the reviews, much in the spirit of social networking websites. Amazon is the largest single source of consumer reviews on the internet. Amazon reviews were collected from the section of joint and muscle pain relief treatments [52].

Mainstream social media provide a generic platform to engage patients. One of their advantages in this context is that many patients are already active users of these platforms, thus effectively lowering barrier to entry to engaging patients online. However, the use of social media in the context of disclosing protected health information may raise ethical issues such as those related to confidence and privacy. The need to engage patients online while fully complying with data protection regulations has led to the proliferation of websites and networks developed specifically to provide a safe space for sharing health-related information online. This systematic review identified 10 platforms of this kind that have been utilized in 21 studies (see Table 6 for details).

Due to ethical concerns, the data used in these studies are usually not released publicly to support further research and evaluation. Only one such dataset has been published. The eDiseases dataset used in 2 studies [53,54] contains patient data from the MedHelp website (see Table 6). The dataset contains 10 conversations from 3 patient communities, allergies, Crohn disease, and breast cancer, which according to a medical expert, exhibit high degree of heterogeneity with respect to health literacy and demographics. The conversations were selected randomly out of those that contained at least 10 user posts. Individual sentences were annotated with respect to their factuality (opinion, fact, or experience) and polarity (positive, negative, or neutral). Annotation was performed by 3 frequent users of health forums. With approximately 3000 annotated sentences with high degree of heterogeneity, this dataset represents a suitable testbed for evaluating SA in the health domain.

**Table 6:** Health-related websites and networks.

Website	Description	Used in
RateMDs [55]	Allows users to post reviews about health care staff and services.	[56-58]
WebMD [59]	Publishes content about health and care topics, including fora that allow users to create or participate in support groups and discussions.	[23,60,61]
Ask a Patient [62]	Allows users to share their personal experience about drug treatments.	[61,63]
DrugLib.com [64]	Allows users to rate and review prescription drugs.	[23,61,63,65]
Breastcancer.org [66]	A breast cancer community of 218,615 members in 81 fora discussing 154,832 topics.	[67,68]
MedHelp [69]	Allows users to share their personal experiences and evidence-based information across 298 topics related to health and well-being.	[21,53,54,70,71]
DailyStrength [72]	A social networking service that allows users to create support groups across 34 categories related to health and well-being.	[23,27]
Cancer Survivors Network [73]	A social networking service that connects users whose lives have been affected by cancer and allows them to share personal experience and expressions of caring.	[74-76]
NHS website [77] (formerly NHS Choices)	The primary public facing website of the United Kingdom’s National Health Service (NHS) with more than 43 million visits per month. It provides health-related information and allows patients to provide feedback on services.	[78]
DiabetesDaily [79]	A social networking service that connects people affected by diabetes where they can trade advice and learn more about the condition.	[80]

As illustrated by the studies discussed thus far, spontaneously generated narrative used in SA typically coincides with the user-generated content, that is, content created by a user of an online platform and made publicly available to other users. The fifth i2b2/VA/Cincinnati challenge in NLP for clinical data [81] represents an important milestone in SA research related to health and well-being. The challenge focused on the task of classifying emotions from suicide notes. The corpus used for this shared task contained 1319 written notes left behind by people who died by suicide. Individual sentences were annotated with the following labels: abuse, anger, blame, fear, guilt, hopelessness, sorrow, forgiveness, happiness, peacefulness, hopefulness, love, pride, thankfulness, instructions, and information. A total of 24 teams used these data to develop their classification systems and evaluate their performance, out of which 19 teams published their results [82-100].

As discussed above, the vast majority of data used in studies encompassed by this review represent user-generated content originating from online platforms. We can differentiate between 2 main types of user-generated content: customer reviews and user comments. A customer review is a review of a product or service made by someone who purchased, used, or had experience with the product or service. The main class of products reviewed in the datasets considered here are medicinal products. Product reviews were collected from Amazon, but also from specialized websites such as Ask a Patient and DrugLib.com. These reviews provide users with additional information about a product’s efficacy and possible side effects typically described in layman’s terms, thus lowering a barrier to participation in health care linked to health literacy and potentially providing better support for shared decision making. Other websites such as RateMDs and the National Health Service (NHS) website allow users to review health care services they received including health care professionals who provide such services. Service reviews can be used by health care providers to identify opportunities to improve the quality of care.

Web 2.0 gave rise to the publishing of one’s own content and commenting on other user’s content on online platforms that provide social networking services. On mainstream social media such as Twitter, Facebook, Instagram, YouTube, and Reddit, patients can organize their fora around groups, hashtags, or influencer users. The primary purpose of these conversations is to exchange information and provide social support online. More specialized websites such as those described in Table 6 serve the same purpose. Spontaneous narratives published on these media represent a valuable source for identifying patients’ needs, especially the unmet ones.

### Data Authors

This section discusses the characteristics of those who authored the types of narratives discussed in the previous section. We first discuss their roles within health and care in relation to research questions RQ3 followed by their demographic characteristics in relation to question RQ4.

We have identified 5 roles with respect to health and well-being among the authors of the types of spontaneously generated narratives considered in this review: sufferer, addict, patient, carer, and suicide victim (see Table 7). Some of these roles may overlap, for example, a sufferer or an addict can also be a patient if they are receiving a medical treatment for their medical condition.

**Table 7:** The roles of authors with respect to health and well-being.

Role	Description	Studies
Sufferer	A person who is affected by a medical condition.	[21,23,27,46,53,54,60,61,63,65,67,68,70,71,74-76,101,102]
Addict	A person who is addicted to a particular substance.	[26,103-106]
Patient	A person receiving or registered to receive medical treatment.	[21,23,27,46,50,53,54,56-58,60,61,63,65,67,68,70,71,74-76,78,80,102,107,108]
Carer	A family member or friend who regularly looks after a sick or disabled person.	[23,56-58,60,61,74-76]
Suicide victim	A person who has committed suicide.	[51,82-100]

Demographic factors refer to socioeconomic characteristics such as age, gender, education level, income level, marital status, occupation, and religion. Most studies involving clinical data summarize the demographics of study participants statistically to illustrate the extent to which its findings can be generalized. Our focus on spontaneously generated narratives implies that the corresponding studies could not mandate the collection of demographic factors. Instead, they can only rely on information provided by users in good faith. Different Web platforms may record different demographic factors, which may or may not be accessible to third parties. Nonmandatory user information will typically give rise to missing values. Moreover, demographic information is difficult to verify online, which raises the concerns over the validity of such information even when it is publicly available.

Table 8 states which demographic factors, if any, are recorded when a user registers an account on the given online services and which ones are accessible online. Only age and gender are routinely collected, but not necessarily shared publicly. Therefore, it should be noted when SA is used to analyze such data to address a clinical question, then the findings should be interpreted with caution as it may not be possible to generalize them across the relevant patient population. Out of 86 studies considered in this review, only 4 reported the demographics factors, [49,67,101,103]. Age was discussed in 3 studies [67,101,103], whereas gender was analyzed in 2 studies [49,103].

**Table 8:** Recording and accessing demographic factors.

Platform	Age	Gender	Education level	Income level	Marital status	Occupation	Religion	Used in
Twitter	?^a^/U^b^	?/N^c^	X^d^/N	X/N	X/N	X/N	X/N	[20-45]
Facebook	M^e^/U	M/U	?/U	X/N	?/U	?/U	?/U	[46,47]
Instagram	M/U	M/U	X/N	X/N	X/N	X/N	X/N	[48]
YouTube	M/U	?/U	X/N	X/N	X/N	X/N	X/N	[49,50]
Reddit	X/N	X/N	X/N	X/N	X/N	X/N	X/N	[51]
Amazon	X/N	X/N	X/N	X/N	X/N	X/N	X/N	[52]
RateMDs	X/N	X/N	X/N	X/N	X/N	X/N	X/N	[56-58]
WebMD	M/U	?/U	X/N	X/N	X/N	X/N	X/N	[23,60]
Ask a Patient	M/Y^f^	M/Y	X/N	X/N	X/N	X/N	X/N	[61,63]
DrugLib.com	M/Y	M/Y	X/N	X/N	X/N	X/N	X/N	[23,61,63,65]
Breastcancer.org	M/U	?/U	X/N	X/N	X/N	?/U	X/N	[67,68]
MedHelp	?/U	M/U	X/N	X/N	X/N	X/N	X/N	[21,53,54,70,71]
DailyStrength	M/U	M/U	X/N	X/N	X/N	X/N	X/N	[23,27]
Cancer Survivors Network	?/U	?/U	X/N	X/N	X/N	X/N	X/N	[74-76]
NHS^g^ website	?/U	?/U	?/U	X/N	X/N	X/N	X/N	[78]
DiabetesDaily	?/U	?/U	X/N	X/N	X/N	?/U	X/N	[80]

^a^? indicates optional recording.

^b^U: user-specific access.

^c^N: not accessible online.

^d^X: recording not available.

^e^M: recording mandatory.

^f^Y: accessible online.

^g^NHS: National Health Service.

### Areas and Applications

This section focuses on the areas of health and well-being encompassed by the given datasets in relation to research question RQ5. These areas provide context for the practical applications of SA, which are discussed in relation to question RQ6.

Support groups provide patients and carers with practical information and emotional support to cope with health-related problems. An ability to record these conversations online offers an opportunity to study and measure unmet needs of different health communities. These communities tend to form around health conditions with high severity and chronicity rates. Not surprisingly, SA has been used to study communities formed around cancer, mental health problems, chronic conditions from asthma to multiple sclerosis, pain associated with these conditions, eating disorders, and addiction (see Table 9 [109-112]). Studying the opinion expressed in spontaneous narratives offers an opportunity to improve health care services by taking into account unforeseen factors. For example, the content of social media can be used to continually monitor the effects of medications after they have been licensed to identify previously unreported adverse reactions [27]. Similarly, SA can be used to differentiate between suicidal and nonsuicidal posts, after which a real-time online counseling can be offered [51].

The provision of health care services itself has been the subject of SA. Table 10 outlines different treatments and services discussed by patients whose opinions have been studied by means of SA. Patient reviews of specific medications can support their decision making but can also be explored to support shared decision making, ultimately influencing health outcomes and health care utilization. Patient reviews of health care services can reveal how the services are experienced in practice [20,56-58,78,107,108,113], help improve communication between patients and health care providers, and identify opportunities for service improvement, again influencing health outcomes and health care utilization. In terms of disease prevention, it is important to understand potential obstacles to population-based intervention approaches such as vaccination [25,32,33,110]. Patients’ opinions can help health practitioners gain insight into the reasons why some patients may opt for traditional and complementary medicine [109]. Alternatively, understanding patients’ experience with different treatments can support creation of personalized therapy plans [45]. SA can be used to continually monitor online conversations to automatically create alerts for community moderators when additional support is needed [60,74]. Practical support can be provided by making online health information more accessible [53,54]. In particular, such information can help carers provide better care to patients [70].

**Table 9:** Health-related problems studied by sentiment analysis.

Problem	Studied in
Cancer	[44,45,75,109], oral [110], lung [71], breast [53,54,67,68,70,71,74,76], cervical [110], prostate [21], colorectal [30,74,76], and cancer screening [38]
Mental health	[34], depression [47,48,111], suicide [51,82-100], and dementia [40]
Chronic condition	diabetes [41,43,44,60,71,80], Chron's disease [46,53,54], multiple sclerosis [22], and asthma [101]
Eating disorder	obesity [36] and anorexia [49]
Addiction	smoking [103-106] and cannabis [26]
Pain	[24,52], fibromyalgia [35]
Infectious diseases	Ebola [28] and latent infectious disease [37]
Quality of life	[29,42,112]

**Table 10:** Health care treatments studied by sentiment analysis

Treatment	Studied in
Medication	[23,27,46,61,63,65,102]
Vaccine	[25,32,110]
Surgery	[114]
Orthodontic	[39,50]
Physician	[56-58]
Health care	[20,31,78,107,108,113]

### Methods Used for Sentiment Analysis

This section studies a range of methods and their implementations that have been used to perform SA in relation to research question RQ7. We also describe their classification performance to establish the state of the art in relation to question RQ8. SA requires an algorithm to classify sentiment associated with narrative text. Typically, sentiment is considered to be positive, negative, or neutral. Therefore, the problem of SA can be defined as that of multinomial classification. When an order can be imposed on the considered classes, then SA can be viewed as an ordinal regression problem.

Traditionally, lexicon-based SA methods classify the sentiment as a function of the predefined word polarities [28,31,37,43,50]. Lexicon-based methods are the simplest kind of rule-based methods. In general, rather than focusing on individual words, rule-based methods focus on more complex patterns, typically implemented using regular expressions [85,87,88,90,93-95,100,112]. Most often, these rules are used to extract features pertinent to SA, whereas the actual classification is based on machine learning algorithms. Table 11 provides information about specific machine learning algorithms used. Specific implementations of these algorithms that were used to support experimental evaluation are listed in Table 12.

To establish the state of the art, we summarized the performance of different classification algorithms in Tables 13 and 14. The results are provided in chronological order. Classification performance measures reported include accuracy (A), precision (P), recall (R), and F-measure, which are calculated using true positives (TP), true negatives (TN), false positives (FP), and false negatives (FN) in the following manner:

A=(TP+TN)/(TP+FP+TN+FN),

P=TP/(TP+FP), R=TP/(TP+FN), F=2PR/(P+R)

**Table 11:** Machine learning algorithms used in sentiment analysis related to health and well-being.

Algorithm	Description	Used in
Support vector machine	Builds a classification model as a hyperplane that maximizes the margin between the training instances of 2 classes.	[25,26,32,33,47,53,67,76,78,82-89,91,92,95,97,98,106,107,110,114]
Naïve Bayes classifier	A probabilistic classifier based on Bayes theorem and an assumption that features are mutually independent.	[26,28,32,38,53,60,61,63,78,93,94,97,98,106,107,114]
Maximum entropy	A probabilistic classifier based on the principle of maximum entropy.	[61,63,67,96,98]
Conditional random fields	A method for labeling and segmenting structured data based on a conditional probability distribution over label sequences given an observation sequence.	[85,98]
Decision tree learning	A method that uses inductive inference to approximate a discrete-valued target function, which is represented by a decision tree.	[47,78,87,97,107,111]
Random forest	An ensemble learning method that fits multiple decision trees on various data samples and combines them to improve accuracy and control overfitting.	[32,53]
AdaBoost	AdaBoost combines multiple weak classifiers into a strong one by retraining and weighing the classifiers iteratively based on the accuracy achieved.	[67,74-76]
*k*-nearest neighbors	A nonparametric, instance-based learning algorithm based on the labels of the *k* nearest training instances.	[47,87]
Logistic regression	A method for modeling the log odds of the dichotomous outcome as a linear combination of the predictor variables.	[26,76,99,111]
Convolutional neural network	A feed-forward neural network that learns to extract salient features that are useful for the given prediction task. Convolutions are used to filter features by using nonlinear functions. Pooling can then be used to reduce the dimensionality.	[30]

**Table 12:** Implementations of machine learning algorithms.

Library	Description	Used in
SVM^light^ [115]	An implementation of SVMs^a^ in C.	[88,91,98]
PySVMLight [116]	A Python binding to the SVM^light^ (see above).	[83]
LIBLINEAR (LIBSVM) [117]	Integrated software for support vector classification, regression, and distribution estimation. It supports multiclass classification.	[32,76,82,84-86,89,95,118]
Weka [119]	A Java library that implements a collection of machine learning algorithms.	[20,23,32,53,54,56,60,76,78,93,94,118]
scikit-learn [120]	A Python library that implements a collection of machine learning algorithms.	[51,104,109]
Keras [121]	A high-level neural networks API^b^ written in Python.	[45]
TextBlob [122]	A Python library that supports NLP^c^ and implements a collection of machine learning algorithms.	[45,51]

^a^SVM: support vector machine.

^b^API: application programming interface.

^c^NLP: natural language processing.

**Table 13:** Classification performance.

Study	Algorithm^a^	Accuracy (%)	Precision (%)	Recall (%)	F-measure (%)
[110]	SVM^b^	70	—^c^	—	—
[82]	SVM	—	55.72	54.72	55.22
[83]	SVM	—	—	—	53.31
[84]	SVM	—	49	46	47
[85]	SVM + CRF^d^ + rules	—	60.1	36.8	45.6
[86]	SVM	—	51.9	48.59	50.18
[87]	KNN^e^, *DT*^f^ *+ SVM + rules*	—	49.92	50.55	50.23
[88]	SVM + rules	—	41.79	55.03	47.5
[89]	SVM, *rules*	—	53.8	53.9	53.8
[90]	rules	—	45.98	44.57	45.27
[91]	SVM	—	46	54	49.41
[92]	SVM	—	55.09	48.51	51.59
[93]	NB^g^, rules, *NB + rules*	—	57.09	55.74	56.4
[94]	NB + rules	—	54.96	51.81	53.34
[95]	SVM, *SVM + rules*	—	—	—	50.38
[96]	ME	—	57.89	49.61	53.43
[97]	*SVM + rules*, NB, DT	—	56	62	59
[98]	SVM + NB + ME^h^ + CRF + lexicon	—	58.21	64.93	61.39
[99]	LR^i^	—	51.14	47.64	49.33
[78]	SVM, *NB*, DT, bagging	88.6	—	—	89
[60]	NB	—	—	—	54
[74]	AdaBoost	79.2	—	—	—
[67]	SVM, AdaBoost, *ME*	79.4	—	—	—
[75]	AdaBoost	79.2	—	—	—
[61]	NB, ME, *rules*	—	85.25	65	73.76
[63]	NB, *ME*	—	84.52	66.67	74.54
[25]	SVM	88.6	—	—	—
[76]	SVM, LR, *AdaBoost*	79.2	—	—	—
[26]	*SVM*, NB, LR	—	71.47	66.91	67.23
[107]	SVM, *NB*, DT	—	—	—	84
[114]	*SVM*, NB	—	63	82	73
[28]	*NB*, lexicon-based	—	75.8	74.3	73
[30]	CNN^j^	76.6	73.7	76.6	73.6
[106]	SVM + NB	82.04	—	—	—
[32]	*SVM*, NB, RF^k^	—	68.73	51.42	58.83
[33]	SVM	—	78.6	78.6	78.6
[111]	LR, *DT*	75	76.1	—	—
[38]	NB	80	—	—	—
[41]	N-gram	—	81.93	81.13	81.24
[53]	*SVM***,** NB, RF	—	—	—	82.4
[47]	SVM, KNN, *DT*	—	58	99	73

^a^Where multiple algorithms were compared, the performance of the best performing algorithm is indicated by italic typeset.

^b^SVM: support vector machine.

^c^Not applicable.

^d^CRF: conditional random fields.

^e^k-nearest neighbors

^f^DT: decision tree

^g^NB: naïve Bayes classifier.

^h^ME: maximum entropy

^i^LR: logistic regression.

^j^CNN: convolutional neural network.

^k^RF: random forest.

**Table 14:** Overall classification performance.

Aggregated value	Accuracy (%)	Precision (%)	Recall (%)	F-measure (%)
Minimum	70.00	41.79	36.8	45.27
Maximum	88.6	85.25	99	89
Median	79.20	57.89	54.87	54.81
Mean	79.80	61.54	60.23	61.52
Standard deviation	5.39	12.63	14.55	13.15

Although a wide range of methods was used, their performance was rarely systematically tested. According to the *no free lunch* theorem [123], there is no universally best learning algorithm. In other words, the performance of machine learning algorithms depends not only on a specific computational task at hand, but also on the properties of data that characterize the problem. SVMs proved to be the most popular choice (see Table 11), which outperformed naïve Bayes classifier (NB) [26,32,53,97,114,124] and random forest [32,51,53]. On occasion, it was outperformed by other methods, for example, NB [78,107], maximum entropy [67], and decision tree [47].

As it can be seen from Table 13, accuracy is not routinely reported, which makes it difficult to generalize the findings and compare them with SA performance in other domains. Nonetheless, we can observe that accuracy does not fall below 70%. On average, accuracy is around 80%. This is well below accuracy achieved in SA of movie reviews, which is typically well over 90% [125-128]. However, it is not straightforward to attribute these results to the intrinsic differences between the domains and their respective sublanguages because of the different choices in methods used. The methods tested on movie reviews are based on deep learning, whereas the methods tested on health narratives still feature traditional machine learning with only 2 studies using neural networks [30,45]. This may be due to the availability of data. Movie reviews are not only publicly available, but also come ready with annotations in the form of star rating. On the other side, health narratives may contain sensitive information and, therefore, cannot be routinely collected en masse. The fact that deep learning does require large amount of data for training may partly explain the preferences toward different types of methods.

Similarly, deep learning is commonly used to support SA of service and product reviews. However, in these domains, the results are closer to those in health and well-being with just over 80% for service reviews and just below 80% for product reviews [129-132]. The performance still lags behind the state of the art achieved in these 2 domains when measured by F-score, which was found to be below 60% on average and can go as low as 45%. F-measure achieved on service and product reviews was found to be in 70s and 80s, respectively [129,133-135]. In summary, the performance of SA of health narratives is much poorer than that in other domains, but it is yet unclear if this is because of nature of the domain, the size of training datasets, or the choice of methods. In addition to the choice of methods, their performance largely depends on the choice of features used to represent text. To support basic linguistic preprocessing, most studies used Stanford CoreNLP [136] (eg, [23,61,63,88,89,95,96,98,99,113]) and Natural Language Toolkit [137] (eg, [51,67,91,96,107,109]). Both libraries represent general purpose NLP tools, which may not be suitable for processing certain sublanguages [138]. It is worth noticing that only 4 studies explicitly stated the use of word embeddings [30,45,53,54].

### Resources

**Figure 2:**
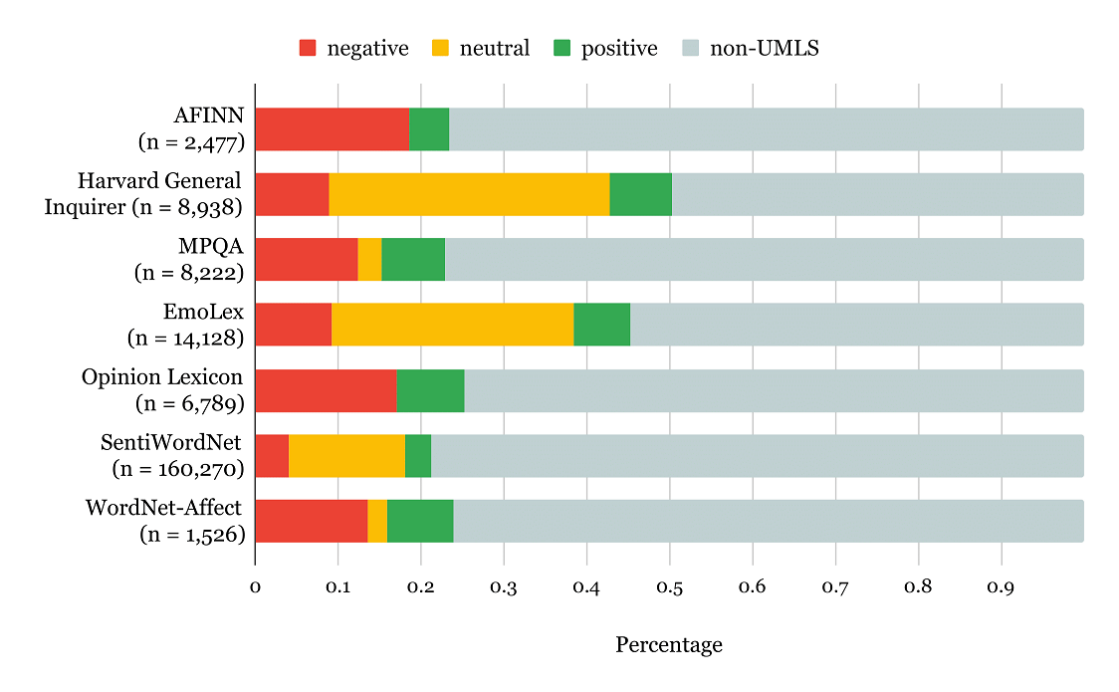
The representation of the UMLS in sentiment lexica.

In relation to research question RQ9, this section provides an overview of practical resources that can be used to support development of SA approaches in the context of health and well-being. Table 15 provides an overview of lexica that were utilized in studies covered by this systematic review. Apart from OpinionKB [61], none of the remaining lexica were developed specifically for applications to health or well-being. To determine how much of their content is specific to health and well-being, we cross-referenced against the Unified Medical Language System (UMLS) [139] using MetaMap Lite [140]. This analysis was limited to publicly available lexica that provide categorical labels of sentiment polarity. The results are shown in Figure 2. On average, 18.55% (with standard deviation of 0.0603) of each lexicon accounts for sentimentally polarized UMLS terms. In relative terms, this accounts for a significant portion of each lexicon given their general purpose. In absolute terms, the number of these terms ranges from as little as 330 in WordNet-Affect to as much as 11,687 in SentiWordNet. Knowing that the UMLS currently contains over 11 million distinct terms, we can observe that at most 1% of its content is covered by an individual lexicon referenced in Figure 2. This means that lexicon-based SA approaches will, by and large, ignore the terminology related to health and well-being.

**Table 15:** Lexical resources for sentiment analysis.

Resource	Description	Used in
Affective Norms for English Words [141,142]	A set of normative emotional ratings for a large number of words in terms of pleasure, arousal, and dominance.	[48,52,89]
AFINN [143,144]	A list of 2477 words and phrases manually rated for valence with an integer between –5 (negative) and 5 (positive).	[24,52,70]
Harvard General Inquirer [145,146]	A lexicon attaching syntactic, semantic, and pragmatic information to words. It includes 1915 positive and 2291 negative words.	[53,54]
LabMT 1.0 [147,148]	A list 10,222 words, their average happiness evaluations according to users on Mechanical Turk.	[31,48]
Multi-Perspective Question Answering [149,150]	A subjectivity lexicon that provides polarity scores for approximately 8000 words.	[27,88,95,105]
Emotion Lexicon (also called EmoLex) [151,152]	A list of words and their associations with 8 basic emotions (anger, fear, anticipation, trust, surprise, sadness, joy, and disgust) and 2 sentiments (negative and positive). The annotations were done manually by crowdsourcing.	[27]
OpinionKB [61,153]	A knowledge base of indirect opinions about drugs represented by quadruples (*e, a, r, p*), where *e* refers to the effective entity, *a* refers to the affected entity, *r* is the effect of *e* on *a*, and *p* is the opinion polarity.	[61]
Opinion Lexicon [5,154]	A list of around 6800 positive and negative opinion words.	[22,27,68,94,112]
SentiSense [155,156]	A lexicon attaching emotional category to 2190 WordNet synsets, which cover a total of 5496 words.	[53,54]
SentiWordNet [157,158]	An extension of WordNet that associates each synset 3 sentiment scores: positivity, negativity, and objectivity.	[23,41,61,63,65,71,83,94,102,113]
WordNet-Affect [159,160]	An extension of WordNet that correlates a subset of synsets suitable to represent affective concepts with affective words. Its hierarchical structure was modelled on the WordNet hyponymy relation.	[85,88,92,94]

Extending the UMLS by including sentiment polarity would address this gap, but this problem is nontrivial as lexicon acquisition has been known to be a major bottleneck for SA. Lessons can be learnt from existing research that focuses on automatic acquisition of sentiment lexicons. These approaches can be divided into 2 basic categories: corpus- and thesaurus-based approaches. Corpus-based approaches operate on a hypothesis that words with the same polarity cooccur in a discourse. Therefore, their polarity may be determined from their cooccurrence with the *seed* words of known polarity [2,161-163]. In this context, MEDLINE [16] would be an obvious source for assembling a large corpus. Similarly, thesaurus-based approaches exploit the structure of a thesaurus (eg, WordNet [164]) to infer polarity of unknown words from their relationships to the *seed* words of known polarity [165-169]. They rely on a hypothesis that synonyms (eg, trauma and injury) have the same polarity, whereas antonyms (eg, ill and healthy) have the opposite polarity. Starting with the *seed* words, the network of lexical relationships is crawled to propagate the known polarity in a rule-based approach. The structure of the UMLS could be exploited in a similar manner to infer the sentiment of its terms.

## Discussion

### Principal Findings

The overarching topic of this review is the SA of spontaneously generated narratives in relation to health and well-being. Specifically, this systematic review was conducted with the aim of answering research questions specified in Table 1. It identified a total of 86 relevant studies, which were used to support the findings, which are summarized here.

### What Are the Major Sources of Data?

The majority of data were collected from the mainstream social multimedia and Web-based retailing platforms. Mainstream social media provide a generic platform to engage patients. However, their use of social media in the context of disclosing protected health information may raise ethical issues. The need to engage patients online while fully complying with data protection regulations has led to the proliferation of websites and networks developed specifically to provide a safe space for sharing health-related information online. This systematic review identified 10 such platforms (see Table 6 for details). In addition to user-generated content, the fifth i2b2/VA/Cincinnati challenge in NLP for clinical data [81] represents an important milestone in SA research related to health and well-being. The corpus used for this shared task contained 1319 written notes left behind by people who died by suicide. This is one of the few datasets that have been made available to research community. Owing to ethical concerns, the data used in the studies included in this systematic review are usually not released publicly to support further research and evaluation. This makes it difficult to benchmark the performance of SA in health and well-being, and test the portability of methods developed. In addition, the lack of sufficiently large datasets prevents the use of state-of-the-art methods such as deep learning (see Tables 12 and 13).

### What Is the Originally Intended Purpose of Spontaneously Generated Narratives?

Web 2.0 gave rise to the self-publishing and commenting on other user’s content on online platforms. On mainstream social media such as Twitter, Facebook, Instagram, YouTube, and Reddit, patients can self-organize around groups, hashtags, and influencer users. The primary purpose of these conversations is to exchange information and provide social support online. More specialized websites such as those described in Table 6 serve the same purpose.

### What Are the Roles of Their Authors Within Health and Care?

We identified 5 roles with respect to health and well-being among the authors of the types of spontaneously generated narratives considered in this review: a sufferer (a person who is affected by a medical condition), an addict (a person who is addicted to a particular substance), a patient (a person receiving or registered to receive medical treatment), a carer (a family member or friend who regularly looks after a sick or disabled person), and a suicide victim (a person who has committed suicide). Some of these roles may overlap, for example, a sufferer or an addict can also be a patient if they are receiving a medical treatment for their medical condition.

### What Are Their Demographic Characteristics?

Our focus on spontaneously generated narratives implies that the corresponding studies could not mandate the collection of demographic factors. Different Web platforms may record different demographic factors, which may not be accessible to third parties. Demographic information is also difficult to verify online, which raises the concerns over the validity of such information even when it is publicly available. Table 8 states which demographic factors, if any, are recorded when a user registers an account on the given online services and which ones are accessible online. Only age and gender are routinely collected, but not necessarily shared publicly. Therefore, any findings resulting from these data should be interpreted with caution as it may not be possible to generalize them across the relevant patient population. Out of 86 studies considered in this review, only 4 reported the demographic characteristics.

### What Areas of Health and Well-Being Are Discussed?

Online communities tend to form around health conditions with high severity and chronicity rates. Not surprisingly, SA has been used to study communities formed around cancer, mental health problems, chronic conditions from asthma to multiple sclerosis, pain associated with these conditions, eating disorders, and addiction (see Table 9). The provision of health care services itself has been the subject of SA. Different treatments and services discussed by patients whose opinions have been studied by means of SA include medications, vaccination, surgery, orthodontic services, individual physicians, and health care services in general.

### What Are the Practical Applications of Sentiment Analysis?

Analyzing the sentiment expressed in spontaneous narratives offers an opportunity to improve health care services by taking into account unforeseen factors. For example, social media can be used to continually monitor the effects of medications to identify previously unknown adverse reactions. Similarly, SA can be used to differentiate between suicidal and nonsuicidal posts, after which a real-time online counseling can be offered. Patient reviews of specific medications can support their decision making but can also be explored to support shared decision making, ultimately influencing health outcomes and health care utilization. Patient reviews of health care services can help identify opportunities for service improvement, thus influencing health outcomes and health care utilization. In terms of disease prevention, patients’ opinions can help health practitioners understand potential obstacles to population-based intervention approaches such as vaccination. Understanding patients’ experience with different treatments can support creation of personalized therapy plans.

### What Methods Have Been Used to Perform Sentiment Analysis?

A wide range of methods have been used to perform SA. Most common choices include SVMs, naïve Bayesian learning, decision trees, logistic regression, and adaptive boosting. Other approaches include maximum entropy, conditional random fields, random forests, and *k*-nearest neighbors. The findings show strong bias toward traditional machine learning. A single study used deep learning. This is in stark contrast with general trends in SA research.

### What Is the State-of-the-Art Performance of Sentiment Analysis?

On average, accuracy is around 80%, and it does not fall below 70%. This is well below accuracy achieved in SA of movie reviews, which is typically well over 90%. In SA of service and product reviews, the results are closer to those in health and well-being with just more than 80% for service reviews and just below 80% for product reviews. However, the performance still lags behind the state of the art achieved in these 2 domains when measured by F-score, which was found to be below 60% on average. F-measure achieved on service and product reviews is found to be above 70% and 80%, respectively. In summary, the performance of SA of health narratives is much poorer than that in other domains.

### What Resources Are Available to Support Sentiment Analysis Related to Health and Well-Being?

A wide range of lexica were utilized in studies covered by this systematic review (see Table 15. Notably, out of 11 lexica, only 1 was developed specifically for a domain related to health or well-being. The lack of domain-specific lexicons may partly explain the poorer performance recorded in this domain.

### Conclusions

In summary, this review has uncovered multiple opportunities to advance research in SA related to health and well-being. Keeping in mind the *no free lunch* theorem, researchers in this area need to put more effort in systematically exploring a wide range of methods and testing their performance. Community efforts to create and share a large, anonymized dataset would enable not only rigorous benchmarking of existing methods but also exploration of new approaches including deep learning. This should help the field catch up with the most recent developments in SA. The creation of domain-specific sentiment lexica stands to further improve the performance of SA related to health and well-being. Although many studies have dealt with automatic construction of domain-specific sentiment lexica using methods such as random walks, no such studies have been identified in this systematic review. Finally, health-related applications of SA require systematic collection of demographic data to illustrate the extent to which the findings can be generalized.

## Abbreviations

API: application programming interface
NB: naïve Bayes classifier
NLP: natural language processing
SA: sentiment analysis
SVM: support vector machine
UMLS: Unified Medical Language System
